# Voices of Hope: Leveraging Think-Aloud Cognitive Interviews to Develop a Hope Assessment Tool for Young People Living with Chronic Health Conditions

**DOI:** 10.3390/children11111396

**Published:** 2024-11-18

**Authors:** Emily von Scheven, Mitchell Braun, Bhupinder Nahal, Emily R. Perito, Paul Brakeman, William Daniel Soulsby, Laura Quill, Addison Cuneo, Linda S. Franck

**Affiliations:** 1 Division of Pediatric Rheumatology, University of California San Francisco, San Francisco, CA 94158, USA; bhupinder.nahal@ucsf.edu (B.N.); daniel.soulsby@ucsf.edu (W.D.S.); 2 School of Medicine, University of California San Francisco, San Francisco, CA 94143, USA; Mitchell.Braun@ucsf.edu; 3 Division of Pediatric Gastroenterology, University of California San Francisco, San Francisco, CA 94158, USA; emily.perito@ucsf.edu (E.R.P.); addison.cuneo@ucsf.edu (A.C.); 4 Division of Pediatric Nephrology, University of California San Francisco, San Francisco, CA 94158, USA; paul.brakeman@ucsf.edu; 5 Division of Pediatric Hematology, University of California San Francisco, San Francisco, CA 94158, USA; quilll@ucsf.edu; 6 School of Nursing, University of California San Francisco, San Francisco, CA 94143, USA; linda.franck@ucsf.edu

**Keywords:** hope, adolescents, think aloud, chronic illness, self-image, identity, questionnaire

## Abstract

Background/Objectives: Hope is a universal, multidimensional, and nuanced concept that may have specific meaning for young people living with chronic health conditions anticipated to last into adulthood. We previously identified definitions of hope for youth living with chronic health conditions derived from young people’s and their caregivers’ own words. Here, we aimed to develop a hope assessment tool to facilitate the future evaluation of interventions to support wellness and health for young people growing up with chronic health conditions; Methods: We developed Likert-type scale questions using the young people’s and caregivers’ definitions of hope and applied the think-aloud cognitive interview method to assess understanding and to inform sequential iteration. Interviews were recorded and insights from participant interviews were analyzed thematically. Results: In total, 11 youth (age 12–16 years) with various chronic health conditions completed surveys and interviews over three iteration cycles. Responses to the six-point Likert-scale questions ranged from 1 (none of the time) to 6 (all of the time) (median 5). All of the young people (n = 11) reported that they do things they enjoy, either all of the time or most of the time. In contrast, only 36% felt energetic, either all or most of the time. Three themes were identified: my body and hope; my identity, self-image, and hope; and my world and hope. Conclusions: In addition to gaining important feedback that allowed us to improve item word choice to maximize assessment tool understanding, we gained valuable insights into the multidimensional construct of hope. Thematic analysis revealed the importance of physical symptoms and identity to the meaning of hope in the context of a young person’s life. Our new hope assessment tool derived from the young people’s own definition of hope has face and content validity for use in clinical and research settings to evaluate hope among pediatric patients living with chronic health conditions.

## 1. Introduction

Hope is a complex construct that is associated with quality of life for individuals living with chronic health conditions [1]. Pediatric chronic disease has become more common and carries the potential for significant negative physical and psychosocial impact on patients, caregivers, and families [2]. In addition to advancing care for underlying diseases, it is important that we develop interventions directed at optimizing health-related quality of life, wellness, and hope. Researchers and healthcare providers often focus on negative states, such as anxiety, depression, and hopelessness. A focus on the positive attribute of hope, informed by the lived experience of the patient community, may facilitate the identification of meaningful interventions with the potential for greater impact [3].

In our previously developed research agenda for youth living with diverse childhood chronic conditions, co-developed with young people and their caregivers, research questions about hope emerged as a priority [4]. In contrast to prior definitions of hope that narrowly focused on overcoming illness and problem-solving to achieve future aspirations, the youth and their caregivers participating in our research characterized hope as a feature of personal identity, and one’s value and potential for impact on the world regardless of the chronic illness. They defined eight features of hope: (1) lightness of spirit, (2) forward looking, (3) vitality, (4) being a person outside of the disease, (5) purpose beyond the basics of care, (6) fulfillment, (7) energy, and (8) aspirations for the future [5]. Exploration of the concept of hope with young people and their families also revealed numerous drivers and facilitators which provide targeted areas for intervention with the potential to make positive change in their lives [5].

Several assessment tools to measure hope in pediatric patients have been developed [6,7,8,9], but they largely do not address the experiences of young people living with a broad range of chronic, treatable, but not curable, conditions. The Children’s Hope Scale [6] assumes that young people are able to achieve their personal goals; however, this may not always be feasible for young people living with chronic health conditions. The children’s version of the Herth Hope Index [7] was developed by adapting questions from an adult questionnaire, and underwent face and construct validation with healthy children, not those living with chronic health conditions. About one in four children in the United States (US) are living with at least one chronic health condition [10]. We hypothesized that a tool derived from the description of hope previously provided by young people with chronic conditions [5] would more accurately and reliably capture the features of hope in the context of their underlying condition than questionnaires constructed by researchers. The current study is an important step in the creation of a validated hope assessment tool for young people living with chronic illness. A hope assessment tool validated across multiple pediatric chronic illnesses can be used to measure hope throughout the course of an illness and help to determine if interventions aimed at increasing hope are effective.

**Figure 1:**
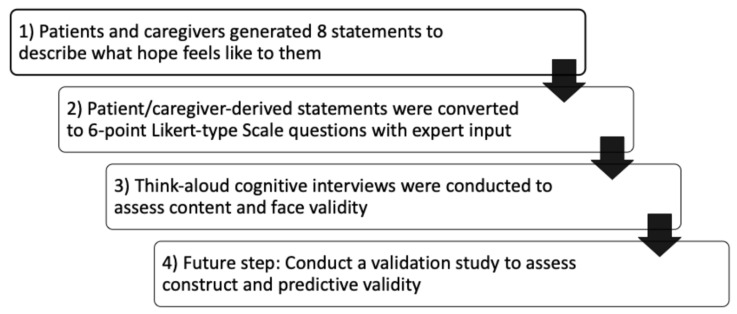
Development and validation of a hope assessment tool for young people living with diverse chronic medical conditions. Results of step 1 were previously published [5]. Results of steps 2 and 3 are presented in this manuscript.

Our overall goal is to develop a tool for assessing hope among youth living with chronic health conditions. In this research, we aimed to develop a tool using the young people and their caregivers’ own definitions of hope and to evaluate its face validity and content validity (Figure 1). The think-aloud cognitive interview method is an effective approach for assessing thought processes and decision-making around a choice in a questionnaire. This method is effective for securing a deeper understanding of an individual’s thought processes, particularly in complex situations [11]. In this report, we describe what we learned from youth using think-aloud qualitative interviews for the development of a new hope assessment tool in young people living with diverse chronic health conditions.

## 2. Materials and Methods

### 2.1. Design

We used a mixed-methods design, iteratively working with youth to develop a hope assessment tool and to test its face and content validity. This work builds on an ongoing collaboration with young people living with chronic medical conditions and their families, where patients and caregivers participate through research activities and advisory councils in the hospital.

### 2.2. Initial Item Selection

The initial version 1 of the assessment tool was created by constructing nine six-point Likert-type response questions from the following eight definitions of hope previously developed by young people and caregivers in our patient community [5]; (1) lightness of spirit, (2) forward looking, (3) vitality, (4) being a person outside of the disease, (5) purpose beyond the basics of care, (6) fulfillment, (7) energy, and (8) aspirations for the future (Table 1). The six response options were the following: all of the time, most of the time, a lot of the time, some of the time, a little of the time, and none of the time. The assessment tool instructions read “Please read each of the statements and think about how you have felt overall in the past 7 days, specifically considering all of the ways that chronic illness is affecting you, then check the box that describes yourself the best. We invite you to honestly express how you are doing!”.

### 2.3. Recruitment and Interviews

Participants were recruited by providers in pediatric subspecialty clinics at a large, urban academic pediatric hospital. Inclusion criteria included English-speaking adolescents who were aged 12–17 years, had self-reported living with a chronic health condition that had lasted over 2 years and was anticipated to last into adulthood, and whose condition resulted in limitations in life or the need for extra medical, psychological, or educational services. We excluded participants who were not able to answer the survey by themselves. This study was approved by the local Institutional Review Board and informed consent was obtained from participants (assent) and their caregivers (consent). Interviews were conducted by two members of the research team (M.B. and B.N.). Interviews were conducted either in person in the clinics or remotely using video conference software (Zoom.us version 6.1.11). Cognitive interviews were conducted with participants using the think-aloud technique [11], in which participants read each statement and explain their answer choices. Probing questions (Table 2) were used to assess understanding according to the following domains: comprehension/interpretation, paraphrasing, confidence, judgment, and recall [12]. Participants were asked about all aspects of the assessment tool, including the instructions. When the participants expressed confusion, interviewers discussed the item and asked the participants to attempt to explain the concept in their own words. The think-aloud interviews ranged from 10 to 20 min long.

### 2.4. Assessment Tool Iteration

The tool was modified in three iterations based on learnings from the cognitive interviews. Each new version was assessed with new participants. The results of the interviews were discussed among the research team and used to inform iterative modifications. Modifications included the removal of questions, the addition of new questions, and revisions to the wording of existing questions. Understandability was used to determine if the wording should be changed, or if new questions should be added. The participant’s report of redundancy in concepts across questions and understandability was used to determine which items to remove. Version 1, which included nine questions, was evaluated by three participants. For version 2, which was evaluated by five participants, three new questions were added for a total of twelve questions. For version 3, which was evaluated by three participants, three more new questions were added for a total of fifteen questions. And for version 4, the final version presented here, six questions were dropped, leaving a total of nine questions (Table 1). Each revised version of the hope assessment tool was evaluated through cognitive interviews in subsequent cycles to confirm readability, understanding, and face validity. New participants were recruited for each iteration cycle, and thus all of the participants did not answer all of the questions across all versions of the assessment tool.

### 2.5. Analysis

Qualitative approaches were used to develop each revised version of the assessment tool. Additionally, qualitative thematic analysis was used to generate a holistic understanding of the participant’s viewpoints and to group participant comments and feedback into themes [14]. The first and last authors listened to the recordings, reviewed the notes for accuracy, and conducted first-level coding to develop themes. Emergent themes were then discussed with the research team and consensus on the final themes was achieved. Quantitative methods were used to assess the participant’s level of hope for each of the items on the assessment tool. Likert-type scale quantitative responses were analyzed using descriptive statistics and graphic visualization. All questions except for “My health condition gets in the way of what I want to do” were phrased positively. Thus, the responses to this question were reverse-coded in the calculation of the total score.

## 3. Results

### 3.1. Participant Characteristics

In total, 11 youths who were living with chronic health conditions each participated in a single think-aloud cognitive interview while completing the assessment tool. Participant ages ranged from 12 to 16 years. Three participants identified as female and eight identified as male; four identified as white and three as Black; and four identified as Hispanic/Latinx. The primary chronic health conditions included rheumatologic (n = 5), gastrointestinal (n = 4), hematologic (n = 2), cardiovascular (n = 1), dermatologic (n = 1), musculoskeletal (n = 1), and psychiatric (n = 1) disorders, with some participants having more than one chronic condition.

### 3.2. Hope Assessment Tool Understanding and Content Validity

The hope assessment tool was modified iteratively three times, following each cycle of interviews. Each cycle was performed with new participants in order to collect fresh input from participants without prior experience of the questions or the think-aloud interview conversation (Table 1). In version 1, the participants reported uncertainty about the meaning of the question “I feel purpose beyond basics of care”. They also expressed different interpretations of the meaning of “basics of care”. Two participants referred to “eating and drinking” and one referred to “the minimum you have to do to take care of yourself”, and asked the researcher for further explanation. There were also differences in understanding of what was meant by “I feel purpose”. One participant described it clearly as “My purpose is more than what is given”. However, the other two were unclear on the meaning. Given the lack of common understanding of some of the questions, in version 2 we added three new questions using more concrete language: “My health gets in the way of what I want to do”, “I am able to balance my health needs and doing what I want to do”, and “I do things I enjoy”.

When evaluating the 12-question version 2, we found that the new questions were easier for the young people to answer. Most participants spoke about the physical impact of their health condition, such as “I need to eat healthy food, but I like to eat fried foods” and the need to “bring medications to [my] friend’s house…but sometimes I forget”. When answering the question “I feel like a person outside of my disease” one participant referred to “not thinking about the disease all of the time, or letting it define you” and another referred to their self-image as it relates to their condition, stating “when people look at me they are not thinking “Oh that is a kid with [disease]””.

Not all participants fully understood what was meant by the word “fulfillment”, so in version 3, we added two additional statements, “I feel useful” and “I do things that are important to me”. Additionally, several participants expressed a lack of clarity about the question “I feel lightness of spirit”, so we added the additional statement “I feel calm and unstressed”. Version 3 included 15 questions. When answering the question about fulfillment, several participants asked for clarification. Those who answered spoke about their social interactions, “in school or with friends and having a good time“, and some spoke about the ability to do things they wanted to do. The statements “I feel lively” and “I feel energetic” were answered similarly by participants. This indicated comprehension and suggested that both questions addressing the same construct were not needed. Due to persistent confusion about some of the original questions, we dropped them in version 4. The nine-question version 4 is presented as our final version and will be further evaluated in future work.

The use of probing questions during the interviews (see Table 2) allowed us to gain further insight and a more nuanced understanding of the participant’s interpretation of the questions, their thoughts, and their experiences. For example, when asked if being useful and doing important things has similar meaning to the idea of fulfillment, one participant explained that “they are [both] part of it, but there is more to it. [I] can feel useful but may not be happy or full. Doing things that are important relates to it…”. Another participant, when asked about fulfillment, answered “Sometimes I get stuck, and then I don’t feel fulfilled”, and another responded, “It can mean different things to different people”. When the interviewer probed to determine if the statement “I look forward to the future” had the same meaning as “I have dreams for my future”, one respondent replied “yes, looking forward means you are moving forward, dreams mean you are going to do something”. These responses, elicited through the use of probing questions, demonstrated the youth’s capacity for nuanced understanding, deep thinking, comprehension of the concept of the future and, for some, abstract thinking.

### 3.3. Usefulness and Feasibility

We solicited feedback on the usefulness of asking questions about hope in the clinical setting and there was agreement by all participants that these questions were relevant and important. One participant commented that the questions “…would be helpful for doctors to know [how] we are feeling so they can understand where we are coming from”. Another participant “liked that it was short, and not too descriptive so it doesn’t make you feel uncomfortable”. Another noted that the questions were “general so [they] can work for a lot of people”. The final version 4 of the assessment tool was felt by the participants to be understandable and feasible to administer to young people using a digital device.

### 3.4. Hopefulness Ratings

**Figure 2:**
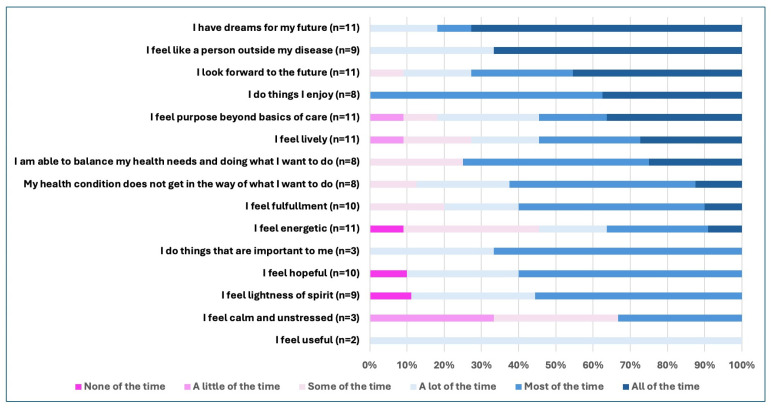
Frequencies of participant ratings for each hope assessment tool question. Number of participants (n) responding to each question varied as some questions were dropped in subsequent versions and others were added.

The hopefulness ratings for the 15 questions assessed ranged from 1 to 6 (median 5) on the six-point scale, where 1 = none of the time, and 6 = all of the time (Figure 2). All of the young people (n = 11) reported that they do things they enjoy, either all of the time or most of the time. Between 70 and 80% of the young people who received the items also responded that they all of the time or most of the time have dreams for their future, are able to balance their health needs with the things they want to do, and look forward to the future. In contrast, just over half of the young people felt lively and that they had purpose beyond the basics of care. Only 36% felt energetic all or most of the time.

### 3.5. Insights into the Lived Experience of Young People with Chronic Illness

The survey responses and the think-aloud interviews revealed important insights into the young people’s lived experiences with respect to their sense of hope. Three main themes emerged from the thematic analysis: “my body and hope”, “my identity, self-image and hope”, and “my world and hope”. The importance of mood and emotional state emerged as important factors throughout the interviews and were prominent across the three themes. Participants used words such as “happy”, “sad”, “feeling up”, “feeling good”, and “believing good things will happen” during the interviews. This highlights the ubiquitous influence of mental health in young people’s lives. The insights from the interviews fell into the following three themes.

#### 3.5.1. Theme #1: My Body and Hope

Participants spoke about the importance of their physical symptoms in determining their sense of hopefulness. Physical symptoms were mentioned both in response to the item “my health condition gets in the way of what I want to do” and in more general items such as “I feel hopeful”. One participant stated “I go day by day. If I am not feeling well, I expect not a good day”. When elaborating on the meaning of the question, “My health gets in the way of what I want to do”, respondents talked about pain, difficulty with strenuous activities, and getting tired when playing sports, pointing to the contribution of the physical experience of their illness to their perception of hope.

#### 3.5.2. Theme #2: My Identity, Self-Image, and Hope

Participants spoke about several aspects of identity and self-image, including their abilities, appearance, and social identity. References to physical appearance included one participant’s description of how their visual appearance caused them to “in school sometimes wear hoodies”, to cover up a rash. Descriptions of ability included getting out of bed, walking normally, attending school, and participating in athletics. References to socialization included both doing things with friends and with family.

#### 3.5.3. Theme #3: My World and Hope

Although the participants mostly shared through the lens of self when describing drivers of hope, including physical, emotional, and social factors, several participants provided a wider perspective. Many referenced family and school as important aspects of their consideration of hope. Several participants shared ideas related to growing up, ”going to college and exploring different places”, “having a job and buying things”, and “leaving home”, demonstrating an ability to see themselves in the context of the larger adult world. One participant drew a distinction between hope for themselves, through the perspective of the individual, and hope for the world, demonstrating the expected developmental progression of increasing incorporation of the perspectives of others as the egocentrism characteristic of adolescence wanes [15].

## 4. Discussion

Hope has been previously defined both as a way of feeling and thinking, and as a factor that impacts one’s behavior. Hope has been studied for decades in individuals with disease [16,17,18]; however, the majority of prior studies investigating hope in childhood illness have derived definitions and theories from studies with adults [7,17]. To our knowledge, this is the first assessment tool measuring hope across a wide range of pediatric chronic illnesses that has been co-developed with young people with lived experience of chronic illness and their caregivers. We used the youth and their caregiver’s own words and definitions to derive the initial version of the assessment tool and made refinements and revisions with input from youth, so that the questions reflect the young people’s understanding of hope in the context of their own lives. In contrast with the questions included in the Children’s Hope Scale [6], which focus on one’s ability to solve problems and comparison with others, the construct of hope presented in this new hope assessment tool includes attributes of physical and emotional state, abilities in the present, and thoughts about the future. In addition to developing this hope assessment tool, we evaluated the face and content validity. With further validation, the tool will provide a standardized approach to measure hope in the way that the chronic illness patient community has defined it [5]. Utilization of this tool in clinical practice will enable the assessment of hope throughout a young person’s healthcare journey and provide useful data for evaluating the effectiveness of interventions designed to increase hope within the setting of chronic illnesses.

In our sample, higher ratings of hopefulness were noted for items related to being able to do enjoyable things. This may reflect the resilience of these young people or where they were in their disease trajectories, as all participants were recruited in the outpatient clinic setting and thus may not have been experiencing disease flares. The low ratings for having energy and feeling hopeful highlight areas of concern and opportunities for intervention. In our prior work, patients and families identified promotors and inhibitors of hope, across several structural, interpersonal, and intrapersonal domains, and proposed three types of interventions to promote hope: resources, navigation, and activities to promote social connection [5]. These provide direction for future research aimed at identifying effective strategies for improving hope. Overall, there was substantial variation in hope ratings represented in this small cohort of youth with chronic conditions. Future research is needed to determine the potential utility of this assessment tool to detect changes over time and responsiveness to interventions aimed at improving hope.

Across the interviews, participants described how their health condition interfered with their physical, emotional, and social well-being and impacted their overall identity. Living with a chronic health condition presents the psychological challenge of needing to integrate one’s identity as an ill person with all the other elements of identity [19,20]. Identity develops through a process of engaging in exploration and integrating how society labels one’s self, all aspects of growing up that can be impacted by living with a chronic health condition [21,22]. The importance of feeling comfortable in one’s body was described by Erikson as an important driver of identity formation [23].

The feedback received through our cognitive interviews using the think-aloud technique highlighted the concrete thinking of young people, consistent with their developmental stage. Piaget [24] described a concrete operational phase of development for young people aged 7–11 years who, although able to grasp the concepts of reversibility and sequencing and able to focus on more than one thing at a time, are still concrete in their thinking. Later, between the ages of 12 and 18 years, adolescents are able to engage with more abstract concepts. Consistent with the concrete, operational phase, many participants in this study were able to consider hope across a broad time frame inclusive of both present and future time points, and even extending into a future state after leaving their childhood home and growing up. The definition of hope includes the element of time, which is perceived differently depending on age, illness, and expected lifespan. Similarly to other hope surveys, our tool does not specify a time frame. Therefore, it is not surprising that participants’ comments ranged from considerations for today to tomorrow, and far into their future adult lives. Several participants demonstrated more abstract thinking and deep nuanced beliefs about the meaning of our questions. However, we found that by replacing some of the more abstract questions with less opaque, more concrete questions during the iteration process, we were able to improve the understandability of the items.

Hope theory has been proposed as being important for behavior change in the field of positive psychology [25,26]. The premise is that higher levels of hope are associated with a greater inclination to set and attain goals. Hope is a driver of agency [25] and has been associated with more positive outcomes, including enhanced self-esteem, self-worth, and self-care agency in both healthy and chronically ill populations [27,28]. Hope is a multidimensional, nuanced concept closely tied to developmental factors and prior experience. Few other hope assessment tools have been published for use with pediatric populations. Snyder et al. in 1997 published the Children’s Hope Scale for use in children aged 8–16 years. Their tool was grounded in the fundamental idea that young people are goal-oriented and understand the means by which they can achieve goals, or “pathways”, and have the ability to initiate and sustain efforts to accomplish goals, through “agency” [6]. However, some have questioned the utility of Snyder’s hope scale, given its one-dimensional focus on goals [7]. Herth and Sarasua (2022) published a children’s version of the Herth Hope Index [8] from the originally developed hope index for adults. Psychometric evaluation was conducted in two phases, first with 22 children recruited from community settings and then with 125 children recruited from community and health clinic settings in the midwestern US. Hinds reported on the development of several hope instruments, including one based on the definition of hope by healthy adolescents and those with substance abuse [29], and a single hope question tool [30,31], which have largely been used in the pediatric cancer population. Our findings suggest that hope for young people is indeed multidimensional. Our hope assessment tool for young people with chronic illnesses has some similarities with previously developed tools, and some unique items.

Most previous works addressing hope have focused on the idea of hoping for a cure and for a return to normalcy. However, a cure is not currently possible for many individuals living with chronic health conditions [32]. We previously published a study conducted with the pediatric chronic illness community focused on identifying the drivers of hope and described three categories of drivers and barriers: (1) structural, (2) interpersonal, and (3) intrapersonal [5]. Structural drivers of hope include healthcare delivery, finances, and research. Interpersonal drivers include community, human connection, and practical support. Intrapersonal drivers include self-development and knowledge, psychological wellness and emotional work, spirituality, and physical wellness. This framework is useful when considering hope, especially within the context of chronic illness, in which individuals interact significantly with the healthcare system and often rely on others to achieve health and wellness [5]. Our new hope assessment tool will be useful in future research and clinical practice to evaluate responses to interventions aimed at modifying the drivers of hope and monitoring changes over time for individuals with chronic illnesses.

There are several limitations to consider in the interpretation of our findings. The sample size is small, and the findings provide only preliminary face and content validity of the new hope assessment tool. Further research with a larger sample is needed to confirm the findings and examine other psychometric properties of the tool. Additionally, the study participants were between the ages of 12 and 16 years, predominately identified as male, spoke English, and primarily lived in San Francisco, California, or the surrounding areas, thus limiting generalizability to other age groups or regions. Similarly, we recruited participants who were able to complete the surveys without assistance and thus our findings are not generalizable to patients with developmental challenges or visual impairment. Recruitment from an out-patient setting may have resulted in patients with better disease control than if we had recruited from an in-patient setting. Thus, the findings of this pilot study with a small sample may not be generalizable to younger patients, non-English speakers, those living in other geographies, or those experiencing a disease flare. Next steps in the psychometric evaluation include direct comparison with other tools to assess convergent and discriminant validity, as well as assessments of responsiveness and predictive validity in longitudinal studies. Another future aim is the development of a version appropriate for younger children.

A major strength of this new nine-item non-disease-specific hope assessment tool is that it was developed with young people and their caregivers, thus demonstrating face and content validity for assessing the elements of hope that are important for youth living with diverse chronic health conditions. The strengths of this study also include the mixed-methods approach and the use of the think-aloud qualitative interview method, which enabled a deeper exploration of the understandability and acceptability of the items.

## 5. Conclusions

Overall, hope is a critical component in healthcare and wellness. We present an assessment tool that can measure hope specifically for pediatric chronic illness. Utilization of the hope assessment tool in clinical settings with interventions designed to increase hope will help clinicians and the healthcare system partner with young people and caregivers to effectively address this important dimension of healthcare. Use of the hope assessment tool in research studies will facilitate the evaluation of interventions and the development of an evidence base for enhancing hope among young people growing up with chronic health conditions.

## Figures and Tables

**Table 1:** Statements included in the four different versions of the assessment tool demonstrating iterative changes in questions based on participant think-aloud cognitive interviews. The assessment tool instructions read “Please read each of the statements and think about how you have felt overall in the past 7 days, specifically considering all of the ways that chronic illness is affecting you, then check the box that describes yourself the best. We invite you to honestly express how you are doing!”). The six-point Likert scale response options were the following: all of the time, most of the time, a lot of the time, some of the time, a little of the time, and none of the time. Each version was completed by different youths.

Version 1 (n = 3)	Version 2 (n = 5)	Version 3 (n = 3)	Version 4 (Final)
I feel hopeful	I feel hopeful	I feel hopeful	I feel hopeful
I feel fulfillment	I feel fulfillment	I feel fulfillment	
		I feel useful	
		I do things that are important to me	I do things that are important to me
I feel purpose beyond basics of care	I feel purpose beyond basics of care	I feel purpose beyond basics of care	
	My health condition gets in the way of what I want to do	My health condition gets in the way of what I want to do	My health condition gets in the way of what I want to do
	I am able to balance my health needs and doing what I want to do	I am able to balance my health needs and doing what I want to do	
I feel lively	I feel lively	I feel lively	I feel lively
I look forward to the future	I look forward to the future	I look forward to the future	I look forward to the future
I feel lightness of spirit	I feel lightness of spirit	I feel lightness of spirit	
		I feel calm and unstressed	I feel calm and unstressed
I have dreams for my future	I have dreams for my future	I have dreams for my future	I have dreams for my future
I feel energetic	I feel energetic	I feel energetic	I feel energetic
I feel like a person outside of my disease	I feel like a person outside of my disease	I feel like a person outside of my disease	
	I do things I enjoy	I do things I enjoy	I do things I enjoy

**Table 2:** Examples of probing questions used by interviewers and participant responses (adapted from Willis [13]).

Type of Cognitive Probe	Survey Question Tested	Probe Question	Example Participant Response
Comprehension	I feel hopeful	What does that mean to you?	“That I can do more than I am doing right now…. that I can accomplish things that I want to”
	I feel fulfillment	Does being useful and doing important things get at the same idea as fulfillment?	“ they are [both] part of it, but there is more to it. Can feel useful but may not be happy or full. Doing things that are important relates to it…”
	I feel calm and unstressed	Do you think calm and lightness of spirit are the same thing?	“…have same meaning but are different…spirit means feel good about yourself…[the] other is just feelings…”
	I feel purpose beyond basics of care	When you hear this phrase, what do you think of?	“if someone needs me …and I am doing something in that moment, then I feel needed.”
	I feel lively	What does this mean to you?	“Doing things that make you feel happy, or like a good person”
Paraphrasing	I feel lightness of spirit	What does it mean in your own words, how would you describe it?	“To be…able to do things, easy going…wanting to try new things…”
Confidence	I feel fulfillment	How sure are you of that answer?	“I don’t know, I feel fulfilment a lot, but there are things not related to my condition that I fail at…”
Recall	I feel hopeful	Why did you choose that answer?	“sometimes I feel sad or hopeless…my pain can be really bad….”
General	I feel fulfillment	Was that easy or hard to answer?	“…harder, but still pretty easy”
	My health condition gets in the way of what I want to do	You answered that quickly, was it easy?	“Yes. Sometimes during PE my feet …hurt, and I need to sit out”

## Data Availability

The de-identified data that support the findings of this study are available from the corresponding author, [E.v.S.], upon reasonable request.
